# Spelling Errors in Brief Computer-Mediated Texts Implicitly Lead to Linearly Additive Penalties in Trustworthiness

**DOI:** 10.3389/fpsyg.2022.873844

**Published:** 2022-05-06

**Authors:** Harry J. Witchel, Christopher I. Jones, Georgina A. Thompson, Carina E. I. Westling, Juan Romero, Alessia Nicotra, Bruno Maag, Hugo D. Critchley

**Affiliations:** ^1^Department of Neuroscience, Brighton and Sussex Medical School, Brighton, United Kingdom; ^2^Department of Primary Care and Public Health, Brighton and Sussex Medical School, Brighton, United Kingdom; ^3^Faculty of Media and Communication, Bournemouth University, Bournemouth, United Kingdom; ^4^Dalton Maag Ltd., London, United Kingdom

**Keywords:** spelling errors, typographic errors, orthographic errors, writing mechanics, trustworthiness, credibility

## Abstract

**Background:**

Spelling errors in documents lead to reduced trustworthiness, but the mechanism for weighing the psychological assessment (i.e., integrative versus dichotomous) has not been elucidated. We instructed participants to rate content of texts, revealing that their implicit trustworthiness judgments show marginal differences specifically caused by spelling errors.

**Methods:**

An online experiment with 100 English-speaking participants were asked to rate 27 short text excerpts (∼100 words) about multiple sclerosis in the format of unmoderated health forum posts. In a counterbalanced design, some excerpts had no typographic errors, some had two errors, and some had five errors. Each participant rated nine paragraphs with a counterbalanced mixture of zero, two or five errors. A linear mixed effects model (LME) was assessed with error number as a fixed effect and participants as a random effect.

**Results:**

Using an unnumbered scale with anchors of “completely untrustworthy” (left) and “completely trustworthy” (right) recorded as 0 to 100, two spelling errors resulted in a penalty to trustworthiness of 5.91 ± 1.70 (robust standard error) compared to the reference excerpts with zero errors, while the penalty for five errors was 13.5 ± 2.47; all three conditions were significantly different from each other (*P* < 0.001).

**Conclusion:**

Participants who rated information about multiple sclerosis in a context mimicking an online health forum implicitly assigned typographic errors nearly linearly additive trustworthiness penalties. This contravenes any dichotomous heuristic or local ceiling effect on trustworthiness penalties for these numbers of typographic errors. It supports an integrative model for psychological judgments of trustworthiness.

## Introduction

### Trustworthiness Online

Trustworthiness of online written information can be affected by errors in the paralinguistic features associated with writing performance (e.g., typographic errors), which is often a shorthand for professionalism, expertise, civility or intelligence (Carr and Stefaniak, 2012); such paralinguistic and pragmatic changes are common in computer mediated communication (CMC) (Bieswanger, 2013).

To quantify this phenomenon, we developed an objective method to quantify judgments of trustworthiness implicitly altered by writing performance in computer-mediated environments. The goal of understanding the marginal differences that writing performance makes to such trustworthiness judgments, independent of the content, is to interrogate the cognitive processes underlying how readers assess penalties to trustworthiness (Albuja et al., 2018). Here we focus on how readers in a computer-mediated experiment will intuitively estimate their own penalties in response to increasing levels of typographic spelling errors. Our goal is to determine whether these penalties to trustworthiness are additive, as recently observed with different types of errors (Witchel et al., 2020), or a fast-and-frugal heuristic that is dichotomous. Cognitive heuristics are known to play an important role in judgments of trustworthiness and trust of information in online environments, and this is an expanding area of pragmatics in computer-mediated communication (Metzger and Flanagin, 2013).

In this paper we address two key issues: (1) quantification of the marginal penalties to estimates of trustworthiness of text excerpts when altered by typographic and orthographic errors, and (2) a methodology for testing integrative versus dichotomous heuristic judgments of penalties to trustworthiness in the context of an unmoderated online health forum. We chose to focus on a single topic (multiple sclerosis) for two reasons: (A) it is a scientific topic, so opinion could not be considered “correct,” and (B) by using a single topic we were comparing like-for-like between statements rated by the same participant. We tested lay assessments of statements that were nominal answers to three important questions that the healthy participants were unlikely to know the answers to:

1. Is multiple sclerosis preventable?
2. How risky is Tecfidera as a treatment for multiple sclerosis?
3. Does multiple sclerosis decrease intelligence/IQ?

### Trustworthiness in Computer Mediated Communication

How readers of CMC make judgments about what writing is trustworthy has been extensively studied (Fogg et al., 2003; Rieh and Danielson, 2007; Diviani et al., 2015), although the specific elements that arouse trust are still being categorized (Sun et al., 2019). Different terms such as credibility, trustworthiness and information quality have been used, although there is no clear consensus between authors as to how these differ or overlap. Elements of trustworthiness may be associated with the source (i.e., the authors), the content, or the medium, and source credibility is often divided into three broad categories: expertise/ability, benevolence/loyalty, and integrity, where some researchers explicitly group benevolence and integrity together as trustworthiness (i.e., researchers define judgments of credibility as trustworthiness that also includes judgments of expertise). For readers of online health forums, the comments of fellow sufferers are likely to be judged as benevolent, their experience with the disease is a sign of expertise, and the fact that they have no financial incentives strongly supports integrity.

How readers assess multiple signals interacting remains open to two broad interpretations. (1) Integrative approaches involve each signal (whether positive or negative) contributing in some way mathematically, whether linearly (e.g., addition and subtraction) or non-linearly. These integrative mental assessments are sometimes summarized as cost-benefit approaches (Sun et al., 2019). (2) Opposing integrative approaches are heuristics, in which a few or one signal will come to dominate the effects of all the other potential signals (Tversky and Kahneman, 1974). An example is the take-the-best heuristic (Bröder, 2000; Gigerenzer, 2008), in which a decision between two alternatives is based upon only the most important property between them that differs; for example, when driving an automobile, if a policeman signals your car to stop, you stop, but in the absence of a policeman, you look for dangerous traffic, but in the absence of dangerous traffic, you follow a stop light, but in the absence of a stop light, you follow a stop sign, etc. A take-the-best heuristic for trustworthiness might be expected to produce a ceiling effect, in which either a text is reliable (no errors), or the author is unreliable (flaws are detected). There are many other heuristics besides take-the-best (Gigerenzer, 2008).

### Typographic Errors

For quite some time it has been known that readers make judgments about both the statement and the author’s ability based on paralinguistic cues such as spelling errors (Diederich et al., 1961; Greenberg and Razinsky, 1966; Lea and Spears, 1992). As a linguistic phenomenon, spelling errors fall in the category of writing mechanics because these errors are isolated to writing (Diederich et al., 1961; Lederman et al., 2014). Spelling errors are often divided into (1) typographic errors (due to incorrect fingering during typing), (2) orthographic errors (when the writer does not know the correct spelling), the latter including homonyms (Kyte, 1958; Figueredo and Varnhagen, 2005), and (3) deliberate mannerisms linked to social capital, the medium or platform (Ling et al., 2014; Zelenkauskaite and Gonzales, 2017). Within the study of CMC, spelling errors affect readers’ judgments of professionalism (Carr and Stefaniak, 2012), intelligence, and competence of the author (Lea and Spears, 1992) as well as credibility and trustworthiness of the message (Metzger et al., 2010; Weerkamp and de Rijke, 2012; Lederman et al., 2014; Sun et al., 2019). Typographic errors lead readers to judge the author as having lower writing ability (Kreiner et al., 2002; Figueredo and Varnhagen, 2005) and the writing as less trustworthy. Spelling errors are said to undercut trustworthiness judgments because they signal personality or attitudinal flaws; that is, the errors reflect a lack of either (1) motivation (conscientiousness, attention to detail or objectivity) to be trustworthy (Vignovic and Thompson, 2010; Morin-Lessard and McKelvie, 2017), or (2) intelligence (education, ability, expertise, authority) (Figueredo and Varnhagen, 2005; Lederman et al., 2014).

The degree to which spelling errors affect trustworthiness judgments are not agreed upon and seem to depend upon the context and expectations of the readers. For example, when professional human resources recruiters rate résumés and application forms, the penalty for having five spelling errors is comparable to having much less relevant work experience (Martin-Lacroux and Lacroux, 2017); this study also showed that the penalty for misspelling was reduced if the recruiter’s own spelling ability was weak, and that the penalty had a ceiling effect, such that ten errors had no greater impact than five errors. Finally, patient-readers of online health forums differ in how they claim to assess trustworthiness, with some claiming that errors in writing mechanics imply a lack of professionalism (Lederman et al., 2014; Sun et al., 2019), while others claim that spelling makes no difference when the information is very basic and the author genuinely cares (Lederman et al., 2014).

### Research Aims and Hypotheses

This research aims to further understand how lay participants assess statements about multiple sclerosis, particularly with regard to additive penalties. We have chosen a scientific topic where (A) the information matters, (B) there should be correct or wrong answers, rather than simply opinions, and (C) the topic should be unfamiliar to the majority of readers, so that the experiment will maximize the effects of paralinguistic features. We hypothesize that in a simulated online environment (H1) participants would judge statements about a scientific topic that they did not know well as significantly less trustworthy if the statements incorporated misspellings, (H2) that the penalties to trustworthiness would be of a similar degree to marginal differences elicited by changes in meaningful content, and (H3) that the penalties would be additive and linear with increasing numbers of errors.

## Materials and Methods

### Participants and Ethical Approval

This experiment was approved by our local ethics committee (Brighton and Sussex Medical School Research Governance and Ethics Committee) and was conducted according to the Declaration of Helsinki. 100 UK participants were recruited via the micropayment platform Prolific during September 2019, and were informed that the study was estimated to last 8–10 min, and the payment was GB £1. Participants had to be English-speaking adults (18+), and were informed that vulnerable populations were excluded from taking part, and a short ethics explanation was provided, where the right to withdraw was explained, and a button labeled “I agree” had to be clicked to continue. Only data that was complete was processed, so any participant who simply chose to leave any part of the online questionnaire incomplete was removed from the data.

### Stimuli: Paragraphs

The experimental stimuli were all text excerpts in the form of a question about multiple sclerosis followed by a user-generated response (70–100 words) in the form of a single paragraph. The nominal responses to these questions were the experimental stimulus excerpts being rated. There were three questions that were used as springboards for the responses (see Introduction).

For each question, there were three different responses (stimuli), totaling nine experimental text excerpts, each a separate stimulus. The experimental stimuli were presented in a randomized order with counterbalancing (Qualtrics). In addition, there were two training excerpts that always preceded the experimental stimuli; these were presented as answers to the question, “Are the artificial sweeteners in diet soda bad for people with multiple sclerosis?” These training stimuli simply were presented to allow participants to get a feel for the rating scale and range of trustworthiness, and they were not labeled as different in any way; the data from training excerpts was not included in the analysis of this study. The complete texts for all stimuli are in Supplementary Material 01 (all supplements are downloadable from github on https://github.com/harry-witchel/Typographic). After each text excerpt the participant had to rate the trustworthiness of the text stimulus using an unnumbered horizontal slider (see Supplementary Methods).

### Text Interventions: Typographic Errors

To determine whether increasing typographic errors leads to additive penalties in trust, we researched the most appropriate ways to add such errors into each short excerpt. For each excerpt, we wanted five words that could be misspelled in a way that was natural for typists, and in such a way that the words would be spread throughout the excerpt (rather than being clustered all at the beginning or at the end). The preferred typographic errors should:

1. be quite noticeable
2. remain clear to the reader even when misspelled (e.g., “yu” plainly means “you”)
3. obviously be a misspelling
4. not be a homonym

To make sure that misspelled words were noticeable, short words were preferred, or we placed the misspellings in the first syllable of a multi-syllable word. All misspellings were found to be naturally occurring on the internet, with at least two usages in health-related websites (see Supplementary Material 02). The types of misspellings were:

1. swap one letter for another letter that is next to it on a qwerty keyboard (“pisitive”)
2. leave out a final silent e (“cognitiv”)
3. double a consonant (“esstimate”)
4. double a vowel, or add an extra vowel (“theere”)
5. leave out a vowel (“expsure”)

### Study Design Process

The nine experimental stimuli (P01–P09) and two training stimuli (T01 and T02) were derived from online discussion groups and websites; the original texts were shortened and edited to be more suitable for the goals of this experimental study. A list of sources for the stimulus excerpts is shown in Table 1, and the complete texts from the original websites, showing how the originals were edited into the stimuli used in this study, are shown in Supplementary Material 03.

**TABLE 1:** Original sources for text stimuli.

Code	Brief topic description	Website	Words
T01	Numerous artificial sweeteners	blogspot.com	78
T02	Hoax about artificial sweeteners	quora.com	81
P01	Vitamin D	quora.com	100
P02	Triggers of the immune system	quora.com	81
P03	Epstein Barr Virus (EBV)	medicaldaily.com	92
P04	Small risk of PML	my-ms.org	90
P05	Up there in risk	quora.com	96
P06	Avonex patient	quora.com	74
P07	Programmer’s intelligence	dailystrength.org	89
P08	Half of all people	ms.pitt.edu	71
P09	Mental exercises	dailystrength.org	85

After the initial paragraphs were designed, a short test study involving friends of the experimental team who did not know the function of the study were invited to take the online survey and provide verbal feedback both on the paragraphs, in terms of comprehension, as well as being asked a range of questions about how they responded mentally to the study. These pre-participants were also asked if they had guessed the nature of the study to be about spelling errors. After feedback a few minor changes to the texts were made.

### Study Delivery and Presentation of Questionnaire

The questionnaire was presented from the Qualtrics platform, which allows for secure presentation and collection of online surveys. The questionnaire consisted of (1) a landing page explaining the participant information associated with ethical approval, (2) a demographics page that asked about age, sex, profession and the age they learned to speak English, (3) an instructions page that explained how to use the slider for ratings, and (4) the paragraph stimuli with ratings sliders, which were presented with two training stimuli followed by a randomized order of the nine experimental paragraphs.

The demographics questions were multiple choice (radio buttons), and all included an option “rather not say”. The instructions for the rating task were as follows:

*You are about to rate your own thoughts and feelings about written text. You will be presented with a series of paragraphs in the style of an online health forum for patients suffering from multiple sclerosis, and you will be asked to rate your response in terms of how convincing you find that paragraph on a sliding scale going from untrustworthy through to completely trustworthy. If you find something trustworthy, you would be prepared to act upon it; an untrustworthy statement you would ignore, and a rating in the middle represents information where you would want more proof or confirmation that it is correct.*

*This scale ranges from the most untrustworthy on the far left of the scale, through to the most trustworthy on the far right of the scale. For example, if you read a paragraph and it is completely untrustworthy, you might rate that paragraph as being at the very far to the left of the scale. If you read a paragraph that you feel is very trustworthy then you would rate that somewhere on the far right of the scale.*

*There are no right or wrong answers to this quiz.*

### Study Design, Analysis, and Statistics

The study design was a confirmatory, cross-sectional experiment with a balanced incomplete block design. To gauge sample size (see Supplementary Methods), we estimated that there would be differences between the zero errors control group and five errors group of 15 and SDs of 25 in each group, so with 100 participants making three trustworthiness judgments per group, and an intracluster correlation coefficient of 0.2, we estimated that there would be >99% power to detect a significant difference with significance set at 0.05. A linear mixed effects model was fitted using the “mixed” command in Stata version 16.0. Residuals from the model were checked at the individual and cluster levels for homoscedasticity and normality. Robust standard errors were employed to calculate appropriate *P*-values and 95% confidence intervals due to heteroscedasticity of the residuals (Williams, 2000). Purpose-made scripts in Matlab were used to plot cumulative probability distributions. Reporting standards were according to the TREND checklist (Des Jarlais et al., 2004), which is provided with the Supplementary Materials (Supplementary Material 04).

## Results

### Variation of Trustworthiness Ratings Between Paragraphs

When comparing the trustworthiness ratings of each excerpt in the no error condition, there was a wide spread of values for each excerpt; nevertheless, there were (as expected) differences in the median ratings between various stimuli (see Supplementary Data). The data demonstrate that the rating scale is adequate to capture the average and extreme trustworthiness values for every paragraph, as there are no obvious ceiling/floor effects; this is essential for testing H2.

### Cumulative Probability Distributions Shifted Left by Typographic Errors

**FIGURE 1:**
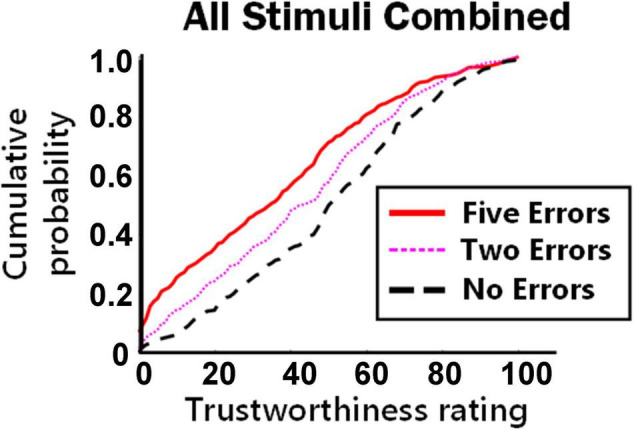
Cumulative probabilities of trustworthiness ratings for all stimulus paragraphs combined. Position of lines toward the lower right of the plot indicates higher trustworthiness compared to lines positioned to the upper left.

To determine the overall effect of different levels of typographic errors on trustworthiness ratings, the cumulative probability distributions were plotted for all ratings combining all text stimuli (Figure 1). As predicted, the distribution for five errors (red continuous line) was consistently upward and to the left (i.e., judged as less trustworthy) than no errors (black dashed line), and the level of trustworthiness for two errors (pink dotted thin line) fell between no errors and five errors. As proposed by H3, this suggests that the penalty to trustworthiness that results from typographic errors is additive (at least between two and five errors) and does not have a dichotomous or ceiling effect for this range of errors. Similar plots were made for each individual text excerpt, showing similar but more variable effects (see Supplementary Data 08).

### Subjective Rationale for Assessment

**FIGURE 2:**
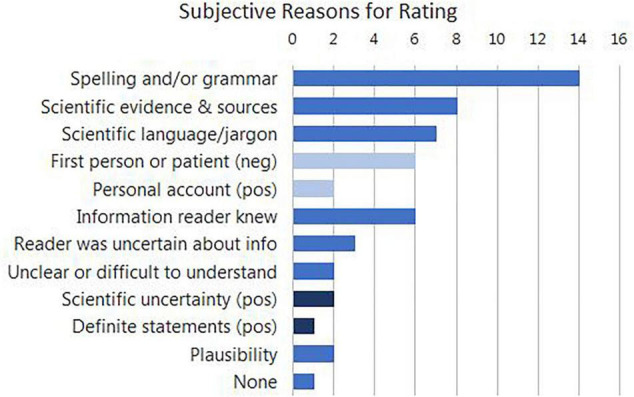
Subjective reasons given for making their ratings. In some cases different participants provided opposing rationales for their judgments (shown in dark and light blue); for example, six participants were negatively influenced by text excerpts that were apparently written by a patient or in the first person, whereas two participants were positively influenced by personal accounts.

To better understand how participants arrived at their ratings, an additional small (30 participant) cohort performed the same experiment with an additional open text question at the end of the survey: “Please explain how you graded the content you read. Were there any issues that influenced you in how you determined any of the ratings you made?” All but one of the participants filled in this box; they provided 1–3 separate reasons, which were categorized as in Figure 2. Nearly half of the respondents specifically mentioned spelling and/or grammar, whereas only 20% of participants mentioned that they made judgments based on information that they previously knew.

### Linear Mixed Effects Model

The data were tested for extent of change and significance using a linear mixed effects model in which the outcome variable was trustworthiness rating and the predictor variables with fixed effects were number of errors (no errors/2 errors/5 errors) and paragraph (see Table 2); the model included a random effect for volunteer number to account for clustering of the data by participant. Paragraph 05 (middle level trustworthiness) with no errors was the reference condition for this model. To allow for the heteroskedasticity of the residuals in this model, robust standard errors were used (Williams, 2000). The intracluster correlation (correlation within the individuals) coefficient estimate is 0.24 (95% CI: 0.17–0.34). This model provides very strong evidence that both two typographic errors and five typographic errors reduce trustworthiness compared to no errors, as predicted by H1. The difference between no errors and two errors was −5.91 units (95% CI: −9.23 to −2.58, *P* < 0.001) and between no errors and five errors was −13.55 units (95% CI: −18.39 to −8.71, *P* < 0.001) on the 100 unit scale. The difference between two errors and five errors was −7.64 (95% CI: 4.12 to 11.16, *P* < 0.001).

**TABLE 2:** Linear mixed effects model for trustworthiness rating (outcome) based on fixed effects of (predictors) error number and paragraph number, with a random effect for volunteer number.

Rating	Coefficient	95% Confidence interval	*P* > | z|
Reference errors: no errors			
Two errors	–5.91	−9.23 to −2.58	<0.001
Five errors	–13.55	−18.39 to −8.71	<0.001
Reference paragraph: para05			
Para01	7.89	1.87 to 13.91	0.010
Para02	–12.92	−18.58 to −7.25	<0.001
Para03	2.20	−3.81 to 8.21	0.473
Para04	15.67	10.51 to 20.83	<0.001
Para06	–2.17	−7.61 to 3.27	0.434
Para07	–9.06	−15.50 to −2.61	0.006
Para08	14.48	8.29 to 20.67	<0.001
Para09	–0.73	−6.66 to 5.20	0.809
_constant	47.66	43.14 to 52.17	<0.001

As predicted by H2, the difference between zero errors and five errors is nearly one half the range of the differences due to statement content; this ranges from Para04 (coefficient = 15.67, 95% CI: 10.51 to 20.83) to Para02 (coefficient = −12.92, 95% CI: −18.58 to −7.25), so the net range is 28.58.

The trustworthiness penalty per error for two errors (penalty = 5.91 ÷ 2 = 2.96) and five errors (penalty = 13.55 ÷ 5 = 2.71) are very close, and suggest that there is a nearly linear relationship between the number of errors and the penalty for trustworthiness. This supports H3.

## Discussion

The novel contribution of this experiment is that healthy volunteers who rated information about multiple sclerosis in a context mimicking an online health forum implicitly assigned typographic errors a nearly linear trustworthiness penalty. While it was well-established in qualitative studies that spelling errors decrease message trustworthiness because of the lack of competence of the author (Singletary et al., 1977; Figueredo and Varnhagen, 2005), it was not clear whether the decrease in trustworthiness was dichotomous (i.e., either competent or incompetent). The results also show that at this level (5 errors in 71 to 100 words) in this context (an experiment mimicking an online health forum), the trustworthiness penalty does not have a ceiling effect. The overarching conclusion is that an integrative model for psychological judgments is a better fit for this data than a heuristic such as “take-the-best.”

Given the high variability between judgments, it is striking that the coefficients for different numbers of errors in the model are linearly related for three reasons. (1) The levels of the model (i.e., no errors/two errors/five errors) were considered as categorical, so the model hypothetically could have led to the conclusion that there was more of a trustworthiness penalty for two errors than for five. (2) We gave the participants no indication that this experiment was about typographic errors. The experiment was advertised and labeled as a test of rating text and emotion, and it asked for a rating of the content of the text. (3) The slider was not numerically labeled, nor did it have tick marks on its axis, so the participants’ rating by positioning of the slider was approximate. Yet, on average people implicitly positioned the difference between two and five spelling errors as linear. As is shown in Figure 2, nearly half of the participants were aware that their judgments incorporated spelling, so this criterion can be considered implicit but not subconscious; it still remains remarkable that lay participants can penalize spelling so accurately whilst incorporating other influences such as the content of the statements.

As can be seen in the Supplementary Data on individual paragraphs (Supplementary Material 08), there is considerable variability in this type of trustworthiness rating data. Not all spelling errors will cause equal penalties to trustworthiness, and this presumably depends on three broad areas and how they relate: the type of error, the reader, and the context or platform. It has been long established that spelling errors can be due to problems with the manual process of typing [or writing (Kyte, 1958)], problems of poor literacy and imperfect knowledge, and as an intentional stylistic device (Zelenkauskaite and Gonzales, 2017). Given the association with poor literacy, spelling was long considered a fundamental flaw for university-bound students, which affects readers’ judgments of form and wording as well as mechanics (Diederich et al., 1961), writing ability, and to a lesser extent, cognitive ability (Kreiner et al., 2002). However, given the possibility of intentional misspelling, the effects of misspelling on judgments of cognitive ability, expertise, or trustworthiness may be complex, reader- and context-dependent. In particular, the quantity of misspelling on unmoderated health forums is so great, especially among adolescents, that it seriously interferes with research (Smith et al., 2014).

It is intuitive that spelling errors in formal situations such as essays and job applications would be associated with a penalty to trustworthiness (Martin-Lacroux and Lacroux, 2017), but it is less obvious why this would be so in emails (Vignovic and Thompson, 2010). Typographic errors may be more common among persons with multiple sclerosis potentially due to issues in the temporo-parietal junction (Carotenuto et al., 2018); therefore, this may impact upon stigma against people with MS online (Rumrill et al., 2015).

### Limitations

By focusing all text excerpts on the topic of multiple sclerosis, it may not be possible to generalize from this topic to all topics. Part of the rationale of this experiment was to understand how lay readers understand scientific information online, so non-scientific information may have different results. Because this study tested healthy lay volunteers about information regarding multiple sclerosis, we anticipated that their estimates of content trustworthiness would be uncertain and thus unduly influenced by paralinguistic and contextual signals; personal descriptions by MS patients may elicit source distrust. It has long been hypothesized that judgments in general, and trustworthiness in particular, are based on two separate pathways, and that the contextual pathway would dominate in the lack of evidence (Petty et al., 1981).

## Conclusion

We conclude that for statements about multiple sclerosis in the context of an unmoderated online health forum, typographic errors elicit a nearly linear trustworthiness penalty in judgments of healthy participants, who would be unfamiliar with the facts of the topic. The objective and unlabeled structure of this experiment leads to a fairly robust evidence on the numerical nature of the effects. From a quantitative point of view, this research leads to three questions: how would the trustworthiness judgments respond to spelling errors made for other subjects (e.g., gardening), how do trustworthiness judgments respond to spelling errors in other contexts (e.g., job applications), and how many spelling errors in this context will it take for the penalties to hit a ceiling effect.

## Data Availability Statement

The datasets presented in this study can be found in online repositories. The names of the repository/repositories and accession number(s) can be found in the article/Supplementary Material.

## Ethics Statement

The studies involving human participants were reviewed and approved by Brighton and Sussex Medical School Research Governance and Ethics Committee ER/BSMS1645/5. The patients/participants provided their online informed consent to participate in this study.

## Author Contributions

HW wrote the ethics application and the first draft and did the preliminary analysis. CJ performed the final statistical models. GT ran the first two series of these experiments. CW developed the stimuli. JR and AN contributed to the design of the initial experiments. BM and HC supervised the work. HW, GT, and HC conceived and designed the research programme. All authors contributed to the article and approved the submitted version.

## Conflict of Interest

BM, AN, and JR were employed by Dalton Maag Ltd. The remaining authors declare that the research was conducted in the absence of any commercial or financial relationships that could be construed as a potential conflict of interest.

## Publisher’s Note

All claims expressed in this article are solely those of the authors and do not necessarily represent those of their affiliated organizations, or those of the publisher, the editors and the reviewers. Any product that may be evaluated in this article, or claim that may be made by its manufacturer, is not guaranteed or endorsed by the publisher.

## Supplementary Material

The Supplementary Material for this article is also available online at GitHub: https://github.com/harry-witchel/Typographic.
