# Supplementary material for: Spelling Errors in Brief Computer-Mediated Texts Implicitly Lead to Linearly Additive Penalties in Trustworthiness
Source: Front Psychol. 2022 May 6;13:873844. doi: 10.3389/fpsyg.2022.873844 (PMC9121982; doi:10.3389/fpsyg.2022.873844)
Supplement: Supplementary file 1 [file Data_Sheet_1.doc]

## Supplementary Methods: Stimulus Paragraphs

## Typographic errors in health forum texts about multiple sclerosis lead to linear penalties in trustworthiness ratings by healthy participants

## (this supplement is available online and is not part of the main published article)

Blue underlined words are misspelled as indicated in versions where misspelling occurs.

### Paragraph P01 - "Vitamin D"

Is multiple sclerosis preventable?

We know that Vitamin D has a prophylactic effect against MS. We know that in countries north of 40 degrees latitude, there is a much higher prevalence of MS, and researchers believe this may be caused by a decrease in exposure to direct sunlight, which is a major cause for vitamin D deficiency.  Studies have also shown us that the vast majority of people who develop MS are deficient in Vitamin D. We know that vitamin D has a prophylactic effect, but we do not know to what extent, how it works, or what level of vitamin D is enough.

4 sentences, 100 words, 152 syllables,

Flesch Reading Ease score 52.868

Misspellings: tht (that), contrys (countries), decrese, defficiency, levell

### Paragraph P02- "Triggers of the immune system"

Is multiple sclerosis preventable?

Yes, MS is completely preventable.  To a large degree, MS is a trigger and response problem. Something triggers the immune system and the immune system responds by attacking the nervous system.  Preventing MS involves being careful to actually reduce exposure to these triggers by, for instance, eating organically grown high quality food, refraining from smoking, using less toxic cleaning products, and basically living a more natural, simpler lifestyle that does not involve constantly bombarding one’s body with reasons to attack itself.

4 sentences, 81 words, 143 syllables

Flesch Reading Ease score 38.5

Misspelling: cmpletely, expsure, insttance, toxxic, connstantly

### Paragraph P03 - "Epstein Barr Virus (EBV)"

Is multiple sclerosis preventable?

The question of MS being preventable has a vague answer.  Multiple sclerosis is said to be hereditary.  Some researchers have linked the Epstein-Barr virus or EBV to multiple sclerosis. Avoiding contact and exposure to this virus might be impossible since the EBV is pervasive. Simple things such proper hand washing and avoiding contact with sick people especially during the cold and flu season may be done to help prevent the acquiring of new EBV.  If you eat more foods that are high in vitamin D, you are more protected against multiple sclerosis.

6 sentences, 92 words, 151 syllables,

Flesch Reading Ease score 53.7

Misspelling: qustion, heraditary, propper, cntact, vittamin

### Paragraph P04 - "Small Risk of PML"

How risky is Tecfidera as a treatment for multiple sclerosis?

There may be a small risk of developing progressive multifocal leukoencephalopathy (PML), which is a rare viral disease of the brain. Three cases of PML have been reported in people taking Tecfidera (dimethyl fumarate). The risk of developing PML on Tecfidera is considered very low but if the immune system is weakened and the body is less able to fight an infection, the virus can reactivate.   A blood test can detect the presence and level of the viral antibodies.  If you are worried, discuss your concerns with your MS team.

5 sentences, 90 words, 146 syllables,

Flesch Reading Ease score 51.325

Misspelling: smalll, peeple, systemm, levell, concernss

### Paragraph P05 - "Up there in risk"

How risky is Tecfidera as a treatment for multiple sclerosis?

Tecfidera is "up there" in the list of MS medicines that carry some moderate to severe risk, as Tecfidera is one of the MS medicines which can cause PML (progressive multifocal leukoencephalopathy).  PML is usually fatal, but survival rates are improving.  One estimate says that the mortality rate is around 30-50% in the first few months after diagnosis. If you do decide to start Tecfidera, make sure you get annual JC virus tests.  Whether or not you have been exposed to the JC virus should factor in to your decision as to which medicine to take.

5 sentences, 96 words, 158 syllables

Flesch Reading Ease score 49.34

Misspelling: theere, wich (which), esstimate, yu (you), meddicine

### Paragraph P06 - "Avonex patient"

How risky is Tecfidera as a treatment for multiple sclerosis?

Beware. I was an Avonex patient for about 16 years. Getting tired of injecting Avonex, I asked my doctor to switch me to tecfidera tablets. Within six months my immune system bottomed out. I had to go off the drug and back on an injectable. It took a good six months or more for my immune system to recover. My neurologist never lost sight or control; he was monitoring my blood levels the whole time.

6 sentences, 74 words, 108 syllables,

Flesch Reading Ease score 67.42

Misspelling: Bewere, paytient, immmune, immmune, monnitoring

### Paragraph P07 - "Programmer's intelligence"

Does multiple sclerosis decrease intelligence/IQ?

I'm positive my IQ has dropped drastically. It use to be 157. My memory was like a steel trap. I was at the top of my game as a programmer. Considered one of the best. Now, they triple check everything I do. I use to have 10 projects going on at the same time. Now they barely give me 2 and I am watched over all the time.  So, I think the answer is “yes”. Next to the pain, I think this is my most devastating symptom. (to me)

11 sentences, 89 words, 117 syllables

Flesch Reading Ease score 87.41

Misspelling: pisitive, steeel, Cnsidered, tripple, baarely

### Paragraph P08 - "Half of all people"

Does multiple sclerosis decrease intelligence/IQ?

Cognitive changes are common in people with MS — approximately half of all people with MS will develop problems with cognition. Cognition refers to a range of high-level brain functions, including the ability to learn and remember information; organize, plan and problem-solve; focus, maintain and shift attention as necessary; understand and use language; accurately perceive the environment; and perform calculations. In MS, certain functions are more likely to be affected than others.

3 sentences, 71 words, 134 syllables

Flesch Reading Ease score 28.60

Misspellings: Cognitiv, apprroximately, nescessary, percive, calculitons

### Paragraph P09 - "Mental Exercises"

Does multiple sclerosis decrease intelligence/IQ?

Do not despair. Many people with MS experience cognitive symptoms like short term memory, ability to quickly process information and so on. Something to think about is doing brain exercises. Every day. There are plenty of websites for brain exercises out there. These sites usually offer some of the exercises free, but charge if you want to track your progress or use other brain enhancing exercises. The websites offer exercises and/or games in thinking flexibility, information processing, memory, math, logic, and so on. Good Luck.

8 sentences, 85 words, 151 syllables

Flesch Reading Ease score 45.76

Misspelling: despear, exprience, wwebsites, exrcises, meemory

## Training Stimulus Paragraphs

These two paragraphs are always presented as the first two stimuli (i.e. before the randomisation of other stimuli). They were never presented with spelling errors or inappropriate capitalization. They are not included in the statistical analyses. The are

### Training Paragraph T01 - "Numerous artificial sweeteners"

Are the artificial sweeteners in diet soda bad for people with multiple sclerosis?

Numerous studies have shown that consuming aspartame can significantly elevate excitotoxins in the blood. When aspartate (as aspartame) is combined in the diet with monosodium glutamate (MSG), blood levels of endotoxins become several fold higher than normal. With the Blood Brain Barrier damaged, as in MS, these excitotoxins can freely enter the site of pre-existing damage, greatly magnifying the damage. A diet high in excitotoxins, such as aspartame, can convert benign, subclinical neural damage into full-blown clinical MS.

4 sentences, 78 words, 148 syllables

Flesch Reading Ease score 30.025

### Training Paragraph T02 - "Hoax about artificial sweeteners"

Are the artificial sweeteners in diet soda bad for people with multiple sclerosis?

There is an online hoax that makes this allegation.   Many people make many claims about aspartame. I like to say that the number one side effect of aspartame is testing in laboratory rats. But aspartame and other sweeteners do not pose any sort of health risk. It is a common scapegoat of fear-mongers.  There is a small chance that artificial sweeteners can have an effect on metabolism that hinders weight loss (and the effect would be minuscule compared to actual sugar).

6 sentences, 81words, 130 syllables

Flesch Reading Ease score 58.84
