# Supplementary material for: Spelling Errors in Brief Computer-Mediated Texts Implicitly Lead to Linearly Additive Penalties in Trustworthiness
Source: Front Psychol. 2022 May 6;13:873844. doi: 10.3389/fpsyg.2022.873844 (PMC9121982; doi:10.3389/fpsyg.2022.873844)
Supplement: Supplementary file 6 [file Data_Sheet_6.doc]

Supplementary methods and results 6 7 8 and 9

For Project:

Spelling errors in brief computer-mediated texts lead to linearly additive penalties in trustworthiness

### Supplementary Methods (06)


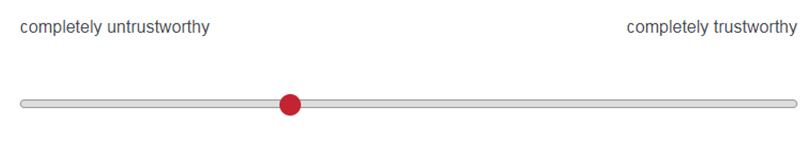


Horizontal slider used for online ratings of trustworthiness. Note the lack of numbers and tick marks.

### Supplementary Data 07


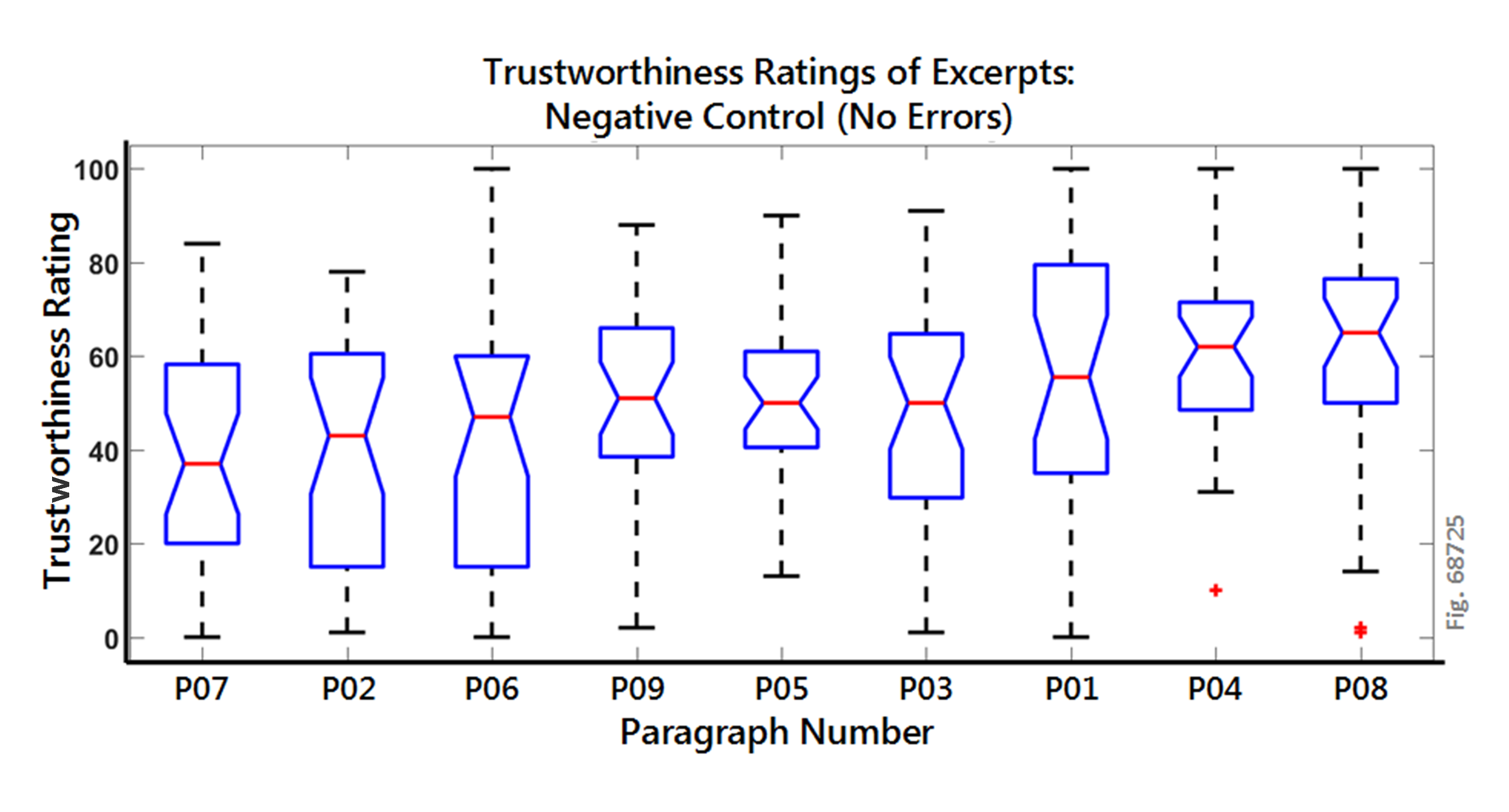


Supplement 07 TKC4RW. Trustworthiness ratings of the stimulus paragraphs when presented without errors. Paragraph numbering is arbitrary and based on their original creation; paragraph presentation order was randomized. Paragraphs are ordered according to trustworthiness ratings for ease of comparison. Outliers (red plus signs) are values > 1.5X the IQR from the lowest quartile.

The box-and-whisker plot shows the median rating for each paragraph (horizontal red lines), the interquartile range (IQR, height of each box), and estimates for the 95% confidence interval (CI) for the median (notches in boxes). The whiskers for each box represent the accepted range for the ratings of each paragraph, although outliers (red plus signs) that extend beyond 1.5X of the IQR show the maximum range. P07 can be considered a low credibility paragraph, P08 is a high credibility paragraph, and P05 can be considered to have a middle credibility.

### Supplementary Data 08


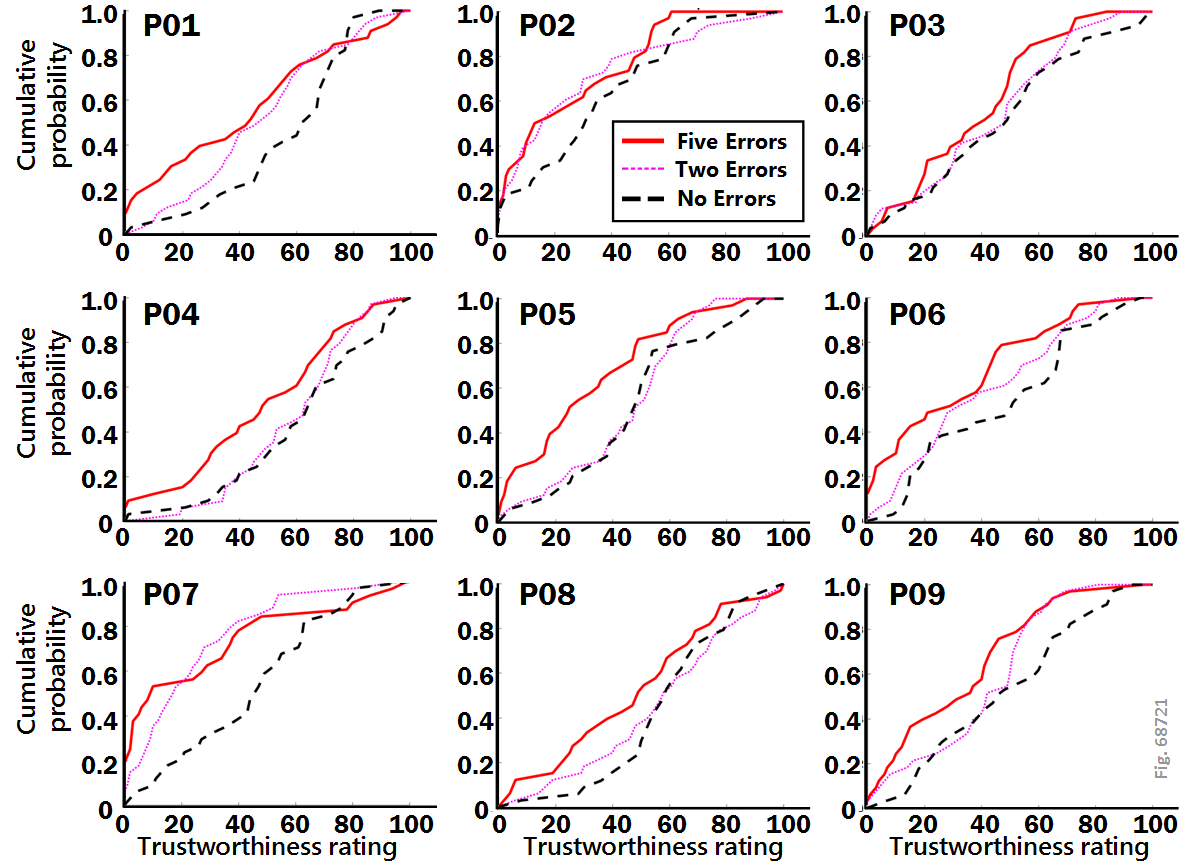


Supplement 08 M32W7. Cumulative probabilities of trustworthiness ratings for each individual stimulus paragraph. Position of lines towards the lower right of the plot indicates higher trustworthiness compared to lines positioned to the upper left.

### Supplementary Methods 09: Power Calculation

We assume that

nA / nB = 1

σ = 25

mean diff = 15

1 - β = 0.99 (strong)

α = 0.05

## Calculate Sample Size Needed to Compare 2 Means: 2-Sample, 2-Sided Equality

This calculator is useful for tests concerning whether the means of two groups are different. Suppose the two groups are 'A' and 'B', and we collect a sample from both groups -- i.e. we have two samples. We perform a two-sample test to determine whether the mean in group A, μAμA, is different from the mean in group B, μBμB. The hypotheses are

H0: μA − μB =0 
H1: μA − μB ≠0

.
where the ratio between the sample sizes of the two groups is 

κ=nA / nB

### Formulas

This calculator uses the following formulas to compute sample size and power, respectively:


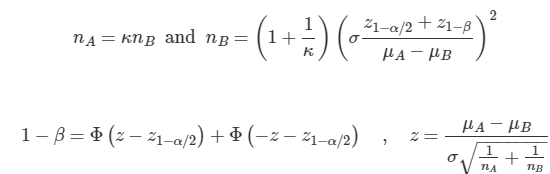


where

- κ=nA/nB is the matching ratio
- σ is standard deviation
- Φ is the [standard Normal](http://en.wikipedia.org/wiki/Standard_normal_distribution) [distribution function](http://en.wikipedia.org/wiki/Cumulative_distribution_function)
- Φ−1 is the [standard Normal](http://en.wikipedia.org/wiki/Standard_normal_distribution) [quantile function](http://en.wikipedia.org/wiki/Quantile_function)
- α is Type I error
- β is Type II error, meaning 1−β is power

### R Code

[R](http://en.wikipedia.org/wiki/R_(programming_language)) code to implement these functions:

| 1  2  3  4  5  6  7  8  9  10 | muA=5  muB=10  kappa=1  sd=10  alpha=0.05  beta=0.20  (nB=(1+1/kappa)*(sd*(qnorm(1-alpha/2)+qnorm(1-beta))/(muA-muB))^2)  ceiling(nB) # 63  z=(muA-muB)/(sd*sqrt((1+1/kappa)/nB))  (Power=pnorm(z-qnorm(1-alpha/2))+pnorm(-z-qnorm(1-alpha/2))) |
| --- | --- |

### References

Chow S, Shao J, Wang H. 2008. Sample Size Calculations in Clinical Research. 2nd Ed. Chapman & Hall/CRC Biostatistics Series. **page 58**.
